# The Effect of Switching From Warfarin to Novel Oral Anticoagulants on Patients’ Satisfaction and the Travel Burden in a Rural Setting

**DOI:** 10.7759/cureus.24608

**Published:** 2022-04-29

**Authors:** Samir Khiralla, Christiaan A Meadows

**Affiliations:** 1 Pharmacology and Therapeutics, Oceania University of Medicine, Apia, WSM; 2 Biochemistry, Oceania University of Medicine, Apia, WSM

**Keywords:** patients satisfaction, vitamin k antagonists, new oral anticoagulants ( noacs), travel burden, warfarin

## Abstract

Introduction: New oral anticoagulants (NOACs) have shown comparable efficacy to warfarin in the treatment of patients with venous thromboembolism (VTE), stroke and atrial fibrillation (AF). Various studies on quality-of-life improvement in rural patients following the switch from vitamin K antagonists (VKAs) to NOACs have produced inconclusive results. The aim of the study is to assess the impact of switching from warfarin to NOACs on remotely living patients’ quality of life and the burden of travel.

Methods: A questionnaire was provided to the patient by their pharmacists. The questionnaire assessed their travel burden and their level of satisfaction with their treatment.

Results: The switch from warfarin to NOACs reduced the burden of travel in 75% of patients. A total of 66% of patients were hesitant about the efficacy of their warfarin treatment. The inconvenience caused due to international normalized ratio (INR) monitoring was reduced in 83% of patients; 70% and 72% of patients strongly agreed that NOACs improved their adherence and treatment satisfaction, respectively. The average number of patients’ travels for INR testing for warfarin monitoring was 7.27 trips/year. The average number of trips made by the patient to obtain their NOACs and warfarin scripts was 2.1 and 4.81 trips/year, respectively.

Conclusion: The switch from warfarin, a VKA, to NOACs in patients who live in remote areas without medical services improved their quality of life, decreased their travel burden, and increased their treatment satisfaction and adherence. Switching to NOACs reduced the number of trips travelled by patients to obtain their anticoagulation scripts and/or to adjust their doses.

## Introduction

Warfarin, a vitamin K antagonist (VKA), was the main oral anticoagulant used for the prevention of venous thromboembolism (VTE) for decades. Recently, the new oral anticoagulants (NOACs) were released to the market and showed potential as a therapy for VTE [1]. In Australia, NOACs that are commonly prescribed include dabigatran, apixaban and rivaroxaban [2].

VKAs reduce the risk of VTE coagulation through the inhibition of the action of vitamin K in the coagulation cascade. On the other hand, dabigatran acts as a direct thrombin inhibitor while rivaroxaban and apixaban act as competitive inhibitors of factor Xa [3]. The usual dosing regime is twice daily for dabigatran and apixaban, but it is once daily for rivaroxaban [1]. In contrast to warfarin that needs international normalized ratio (INR) monitoring, NOACs do not require INR follow-up irrespective of patients’ sex and age [4]. Several studies have found that NOACs have comparable and or superior efficacy to warfarin in the management of VTE or atrial fibrillation (AF) [2,5].

Gebler-Hughes et al. assessed patients’ perception of long-term treatment with NOACs versus warfarin and whether their residential status might affect the transition from warfarin to NOACs. The study revealed that 82% of rural patients and 50.3% of urban patients had not heard about the NOACs. The transition from warfarin to NOACs showed fewer benefits in these cohorts of patients [6].

Keita et al. used three validated questionnaires to evaluate satisfaction, adherence, and quality of life in VTE patients who were using VKAs or who were transferred from VKAs to NOACs and vice versa. The study showed that NOACs are non-superior to VKAs in patients’ satisfaction. Patients who switched from VKAs to NOACs and who used NOACs for at least three months showed the highest adherence. In relation to the quality of life, NOACs were superior to VKAs [7].

Bellinge et al., in a retrospective study, assessed whether or not the distance from the medical services impacted the determination of the anticoagulation treatment. The study found that 29% versus 36% (P = 0.08) had been prescribed warfarin versus NOACs for patients who do not live locally. Moreover, the study showed that NOACs are superior to warfarin in relation to bleeding events in the patients while they are on the anticoagulation regimen (nine events for NOAC therapy vs 20 events for warfarin therapy). The study found that only one-third of the patients eligible for anticoagulation had received NOACs [8].

The purpose of our study is to assess the impact of the switching from warfarin to NOACs on patients who are living in remote Australian areas where medical amenities are not available in their suburbs. Our goal in this research is to evaluate the outcome of this switch on the burden of travel incurred by those patients and their satisfaction after the switch. We expect that the switch from warfarin to NOACs will reduce the travel burden and increase patients’ satisfaction with their treatments. Thus, the present state of knowledge would benefit from further evaluation of the relevance of NOACs for rural patients.

## Materials and methods

This was a survey-based study, and the Oceania University of Medicine (OUM) Institutional Review Board committee granted an approval (no. 20-0619SK). The participants of the study were selected from remote places in Australia where there was no medical practitioner and no pathology services were available to patients to undergo the INR testing. The remote locations included were Ariah Park, New South Wales (NSW); Ardlethan, NSW; and Stanley, Tasmania, Australia.

We included patients who initially received warfarin as an anticoagulant and then switched to NOACs in the last 24 months and had used warfarin for at least six months. Patients were excluded if they have not used warfarin before and were currently taking NOACs. We also excluded patients who were taking warfarin and had never taken NOACs before and patients who switched from warfarin to NOACs more than 24 months ago.

The questionnaire was offered to patients by their attending pharmacist. Informed consent was obtained from the interviewed patients and their anonymity was guaranteed. The pharmacist explained to the patients that there were two sets of questions to be answered. In the first set of questions, patients were asked to choose the answer that best described their perceptions, while in the second set of questions, they were asked to give a numerical answer to the questions. The pharmacists did not intervene with the answers of the patients and the patients were offered a private area in which to answer the questionnaire.

The questionnaire (provided in the Appendix) was designed to assess our hypothesis in the study; it included six Likert style questions and five numerical questions [9,10]. The Likert-based questions were used to address the patient’s estimation of the potential burden of travel and overall satisfaction. The numerical questions were designed to assess the frequency of travel as well as the time spent to obtain the scripts and the frequency of travel to adjust the warfarin dosage. The numerical questions were averaged, and the mean was obtained. The period over which data was collected was three months from September 14, 2020, to December 4, 2020. Data were analyzed and figures were generated using Microsoft Excel 2016 (Microsoft, Redmond, Washington).

## Results

The total number of participants was 53, with 25 male participants (47%) and 28 female participants (53%). The average age of the patients was 69.88 years. After the switch from warfarin, 21 patients received rivaroxaban 20 mg once daily, 30 patients received apixaban 5 mg twice daily, and 2 patients received dabigatran 150 mg twice daily.

**Figure 1:**
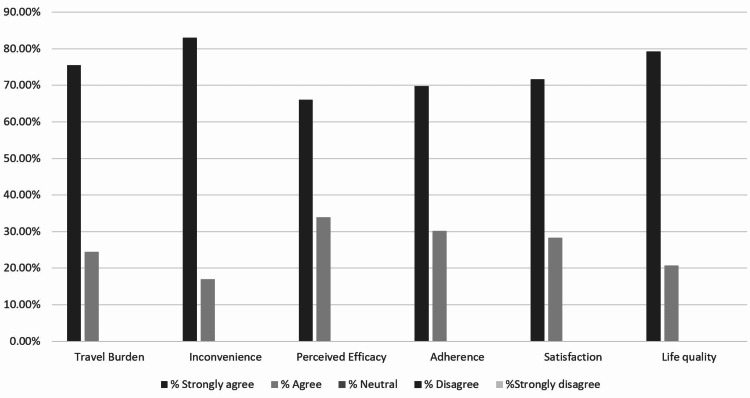
Patients’ perceptions of their experience of switching from warfarin to new oral anticoagulants Travel burden, inconvenience, efficacy, adherence, satisfaction, and life quality represent questions 1 to 6 in the questionnaire, respectively. The black and grey columns represent the percentage of patients who strongly agreed and agreed with the asked question, respectively. Neutral, disagree and strongly disagree choices were not chosen by any patient and they have zero percentages.

The study utilized a questionnaire; therefore, the results are based on patient perceptions. Self-evaluation is always a potential concern or limitation for studies that utilize questionnaires for data collection. In order to further evaluate the patients’ responses against a more quantitative measure, we included the second set of numerical questions. For example, patients were asked whether they felt travel was burdensome owing to duration or frequency in the Likert format questions. The numerical questions obtain data on the actual number and duration of trips. If patients answered that questions about dosage led them to doubt the efficacy of their warfarin treatments, this perception could be compared with the actual number of trips they had to make in order to measure or adjust the warfarin dose. Figure 1 shows patients’ perceptions of their experience of switching from warfarin to NOACs.

In relation to the switching from warfarin to NOACs and their effect on the reduction of the travel burden, 75% versus 25% of the patients strongly agreed and agreed, respectively, that switching to NOACs had reduced their burden of travel.

The data regarding the inconvenience incurred by the travel to adjust the dose of warfarin based on the INR measurement showed that 83% and 17% of the patients strongly agreed and agreed, respectively, that the travel to measure the INR was inconvenient. Patients were asked whether the uncertainty about correct warfarin dosing caused them to question the efficacy of their treatment. We observed that 66% and 34% of the patients strongly agreed and agreed, respectively, that the dose uncertainty caused them to question the treatment efficacy.

**Figure 2:**
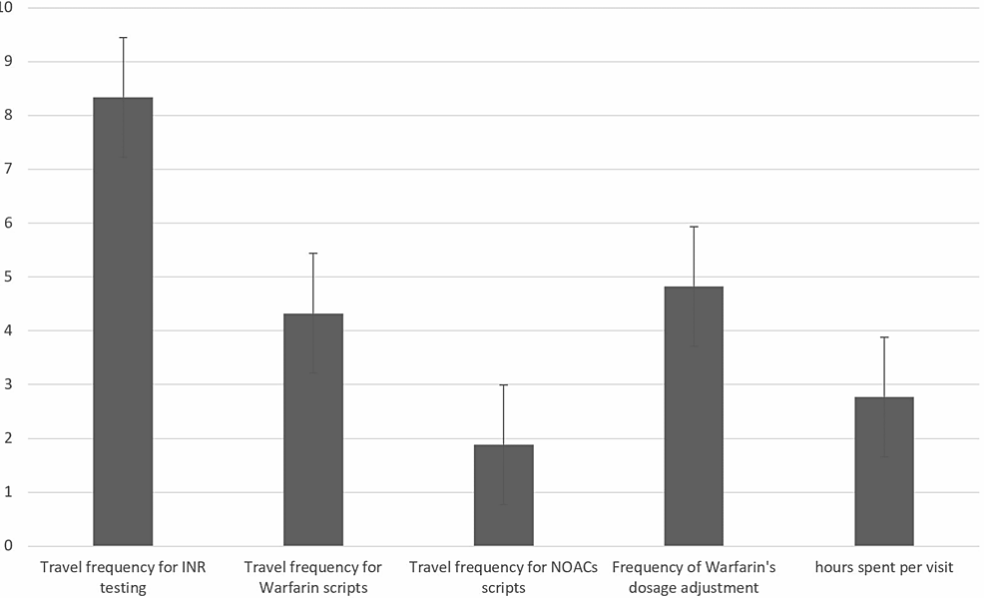
Frequency and duration of travel burden NOACs, new oral anticoagulants The columns in the figure represent questions 7 to 11 in the questionnaire

Assessing the effect of the switch from warfarin to NOACs on the patient’s adherence, 70% of the patients strongly agreed that their adherence was improved by the switch and a further 30% agreed that the switch to NOACs had improved their adherence. We noted that 72% and 28% of the participants strongly agreed and agreed, respectively, that the treatment with a fixed dose had increased their satisfaction with their treatment. Regarding the effect of the NOACs on the improvement of the participant’s quality of life, 72% of the patients strongly agreed that the NOACs had improved their quality of life and 28% of the participants agreed that the new treatment had improved their quality of life. Figure 2 shows the frequency and duration of the travel to obtain scripts and medication dose adjustments.

On average, patients travelled 7.41 times to measure their INR per year and the range of the number of travels was 3-12 times per year. Comparing the number of trips travelled by the patients to obtain warfarin scripts versus NOAC scripts per year, the average number of the trips was 4.9 versus 2.2 trips, respectively. The mean number of times that the patients found that the warfarin dose needed to be changed was five times per year with a range of 2-10 times. The average number of hours spent on travel by different participants per visit was 3.81 hours with a range of two to seven hours per trip.

## Discussion

In rural Australia, where there is a shortage in medical services in a patients’ vicinity, patients need to travel to have their INR measured. Travelling may be considered a burden on the patients and may affect their quality of life. The assessment of whether switching from warfarin to NOACs can reduce the burden of travel showed that 75% of the participants strongly agreed that their burden of travel had been reduced by switching their treatment to NOACs. The remaining 25% of the patients agreed that switching had reduced their burden of travel. Patients used to travel an average of 7.41 trips/year to measure their INR where the minimum number of trips was 3 trips/year, and the maximum number of trips was 12 trips/year. Considering the NOAC dosage does not require such INR monitoring, the high number of patients’ trips/year to monitor their INR can reflect why the switch from warfarin to NOACs reduced the patients’ burden of travel. That finding is augmented by the fact that 83% of the patients strongly agreed that the travel to test for their INR was inconvenient and the remaining patients (17%) agreed that the travel to adjust their INR was inconvenient.

Mearns et al. found that about 61% of the time, the patients treated with VKAs were found out of the optimal range of the INR. The poorer the INR control was, the greater the chances of VTE recurrence were [11]. We expected that the poor control of the INR may affect a patient’s satisfaction with the warfarin treatment. NOAC usage is characterized by a fixed once- or twice-daily dose. This may increase the patient’s satisfaction by reducing the need for dosing visits, especially in such rural settings. Our study assessed the number of times that the patients treated with warfarin had to change their dose. We found that the number of times that the patients had to change their dose ranged from 2 to 10 times per year with the average number of dose changes per year being 5. In relation to the patient’s perception of the uncertainty of the correct warfarin dosing and whether this would cause one to question the treatment efficacy, we found that 66% and 34% of the patients strongly agreed and agreed, respectively, that the dose uncertainty caused them to doubt the efficacy of their treatment. On the other hand, the switch from warfarin to NOACs and receiving a fixed daily dose increased the patient’s satisfaction. We noted that 72% and 28% of the patients strongly agreed and agreed, respectively, that NOACs increased their treatment satisfaction. The finding by Mearns et al. that 61% of the patients had either higher or lower INR values than their optimal values can explain our finding of the patients’ strong agreement about their satisfaction with receiving a daily fixed dose and their uncertainty about the efficacy of their warfarin treatment [11].

Gebler-Hughes et al. concluded that there were no benefits from the transition from warfarin to NOACs. However, 82% of the rural recruited patients had not heard about the NOACs at the time of the study [6]. In our study, the patients strongly agreed that the NOACs had reduced their travel burden and increased their satisfaction with their treatment. Therefore, the absence of awareness of the NOACs may have contributed to the outcome of the Gebler-Hughes et al. study [6].

Keita et al. found that the switch from VKAs to NOACs did not improve patients’ satisfaction [7]. However, in our study, the assessment of patient satisfaction due to switching from warfarin to NOACs demonstrated that the majority of the patients (72%) strongly agreed that the NOACs increased their satisfaction. The finding in our study can be attributed to the unique nature of our rural patients who had no local medical facilities in their suburbs.

In relation to the assessment of patients’ adherence and quality of life upon switching to NOACs, NOACs were found to improve the quality of life of the patients and increase their adherence. A total of 70% and 72% of the participants strongly agreed that the switch to NOACs has improved their adherence and quality of life, respectively. Our findings for the improvement of adherence and quality of life were similar to the finding of Keita et al. [7]. The average number of trips that patients made to obtain their script of warfarin versus NOACs showed 4.9 trips/year versus 2.2 trips/year, respectively; therefore, switching to NOACs reduced the average number of trips by 55.1%. The great decrease in the number of trips to obtain NOAC scripts can be considered another contributor to patient satisfaction and quality of life improvement shown with switching to NOACs. The patients treated with warfarin spent an average of 3.81 hours per visit to obtain their warfarin scripts. The number of hours was variable according to the location of patients and their treating physicians. Considering the average number of hours spent and the number of extra trips made by patients to obtain their scripts or to measure their INR can explain patients’ response to the questionnaire questions.

In Australia, Medicare coverage greatly contributes to the cost of the medications; for example, the cost of NOACs and warfarin on the national Pharmaceutical Benefits Scheme (PBS) is the same for pensioners [12]. In our study, all the patients were pensioners, and they were covered by Medicare Australia; therefore, the cost of the medications has not been addressed in the survey. However, the initial cost of warfarin is cheaper than NOACs, and the cost incurred due to warfarin monitoring increases the overall treatment cost [13]. In such rural settings, the monitoring cost may be higher than in urban settings.

Our study’s main limitation was the small population in the remote areas of Australia. Future studies on more locations may include a larger number of participants. Moreover, studies assessing the effectiveness of a treatment strategy or plan in the future might need to account for travel burdens in remote populations.

## Conclusions

The switch from warfarin to NOACs by rural Australians who did not have medical services in their vicinity improved their quality of life, adherence to their medication and increased their satisfaction with their treatment. Patients who were treated with warfarin showed high hesitancy about the efficacy of their treatment and they needed to adjust their warfarin dose frequently per year. Switching to NOACs reduced the number of trips made by patients by eliminating the need for INR measurement and by reducing the frequency of trips to obtain scripts for anticoagulants and/or their dose adjustment. Patients' perceptions of the switch were found to be unanimously positive. The numerical data both support patient perceptions and provide a plausible explanation.

## Appendices

Questionnaire

1) The switching to NOACs has reduced my burden of travel.

             Strongly disagree           disagree             neutral          agree              strongly agree

2) Travel to adjust the dose of the INR/ warfarin dose was inconvenient

              Strongly disagree           disagree             neutral          agree              strongly agree

3) Uncertainty about correct dosing causes me to question the efficacy of treatment

             Strongly disagree           disagree             neutral          agree              strongly agree

4) The switch from warfarin to NOACs has increased my adherence

               Strongly disagree           disagree             neutral          agree              strongly agree

5) Being on a fixed daily dose has increased my satisfaction with the treatment.

               Strongly disagree           disagree             neutral          agree              strongly agree

6) The new treatment has improved my quality of life

                Strongly disagree           disagree             neutral          agree              strongly agree The second set of questions was intended to attain numerical data on the frequency and duration of travelling to assess the time and difficulty involved.

7) How many times did you travel to test for INR/year?

8) How many times did you travel to get your scripts for warfarin/year?

9) How many times did you travel to get a script for apixaban, rivaroxaban, or dabigatran/year?

10) How many times per year did you find the warfarin dose needs to be changed?

11) How many hours did you spend on travel per visit?
